# Trop-2 is up-regulated in invasive prostate cancer and displaces FAK from focal contacts

**DOI:** 10.18632/oncotarget.3960

**Published:** 2015-04-29

**Authors:** Marco Trerotola, Kirat K. Ganguly, Ladan Fazli, Carmine Fedele, Huimin Lu, Anindita Dutta, Qin Liu, Tiziana De Angelis, Luke W. Riddell, Natalia A. Riobo, Martin E. Gleave, Amina Zoubeidi, Richard G. Pestell, Dario C. Altieri, Lucia R. Languino

**Affiliations:** ^1^ Prostate Cancer Discovery and Development Program, Thomas Jefferson University, Philadelphia, PA, USA; ^2^ Department of Cancer Biology, Sidney Kimmel Cancer Center, Thomas Jefferson University, Philadelphia, PA, USA; ^3^ The Vancouver Prostate Centre, University of British Columbia, Vancouver, British Columbia, Canada; ^4^ Tumor Microenvironment and Metastasis Program, The Wistar Institute Cancer Center, Philadelphia, PA, USA; ^5^ Department of Biochemistry, Thomas Jefferson University, Philadelphia, PA, USA; ^6^ Current address: Ce.S.I. – University of Chieti-Pescara, Chieti Scalo, Italy

**Keywords:** pT2/pT3/pT4 prostate cancer, metastasis, gleason grade, TRAMP, exosome

## Abstract

In this study, we show that the transmembrane glycoprotein Trop-2 is up-regulated in human prostate cancer (PCa) with extracapsular extension (stages pT3/pT4) as compared to organ-confined (stage pT2) PCa. Consistent with this evidence, Trop-2 expression is found to be increased in metastatic prostate tumors of Transgenic Adenocarcinoma of Mouse Prostate mice and to strongly correlate with α5β1 integrin levels. Using PCa cells, we show that Trop-2 specifically associates with the α5 integrin subunit, as binding to α3 is not observed, and that Trop-2 displaces focal adhesion kinase from focal contacts. In support of the role of Trop-2 as a promoter of PCa metastatic phenotype, we observe high expression of this molecule in exosomes purified from Trop-2-positive PCa cells. These vesicles are then found to promote migration of Trop-2-negative PCa cells on fibronectin, an α5β1 integrin/focal adhesion kinase substrate, thus suggesting that the biological function of Trop-2 may be propagated to recipient cells. In summary, our findings show that Trop-2 promotes an α5β1 integrin-dependent pro-metastatic signaling pathway in PCa cells and that the altered expression of Trop-2 may be utilized for early identification of capsule-invading PCa.

## INTRODUCTION

The molecular mechanisms underlying the early phases of tumor invasion are not completely understood, although it is largely believed that acquisition of enhanced capacity to migrate through the extracellular matrix (ECM) is a critical step for the onset of the metastatic cascade. When diagnosed at a non-invasive stage, prostate cancer (PCa) is generally curable by surgical removal of the prostate gland. However, when PCa cells acquire the ability to break through the external capsule and invade the surrounding tissues, the chances to eradicate the disease by radical prostatectomy are reduced, resulting in lower overall survival rates for patients with metastatic disease. Widely accepted tumor staging criteria establish that stage pT2 identifies PCa still confined within the prostatic gland, whereas stages pT3/pT4 refer to PCa that has spread through the capsule and has eventually invaded adjacent structures [1, 2]. Traditional disease monitoring approaches, including circulating prostate specific antigen (PSA) levels or Gleason scoring, do not discriminate between stage pT2 and stages pT3/pT4 [1, 2], thus hampering a central tenet for cancer patient stratification [3]. Therefore, developing molecular biomarkers that could identify specific stages of PCa progression remains an urgent, unmet medical need.

Previous studies have reported that the expression profile of many integrins, receptors for ECM substrates, becomes aberrant during cancer progression [4, 5]. In particular, the α5β1 integrin heterodimer plays a pivotal role in development and progression of several types of carcinomas, including PCa [6, 7], and its expression correlates with reduced disease-free survival in several malignancies [7-9]. The α5β1 integrin is implicated in cell proliferation and growth [10]. A function-blocking antibody against α5β1 integrin significantly reduces tumor burden and metastasis in ovarian cancer models [7]. Additional studies demonstrate that the α5β1 integrin directly supports cell migration/invasion and metastasis [11, 12].

Metastatic dissemination is also promoted by exosomes, vesicles of endosomal origin, which are believed to generate a suitable microenvironment in the pre-metastatic niche [13, 14] by mediating horizontal transfer of genetic material [15] as well as of signaling molecules [14].

The epithelial transmembrane glycoprotein Trop-2 functions as a key regulator of β1 integrin activities by inducing cell detachment from ECM substrates and promoting motility of PCa cells [16, 17]. Trop-2 overexpression has been consistently linked to poor prognosis in many human cancers [18-21], suggesting a potential role of this molecule in metastatic dissemination. Specifically, we have previously shown that Trop-2 inhibits localization of β1 integrins in focal adhesions (FAs) and induces hyperphosphorylation of focal adhesion kinase (FAK), deregulating cell-ECM interactions [17].

Altogether, the experimental findings presented here show that Trop-2 is a novel marker of capsule-invasive PCa, is found in PCa cell exosomes and may function as mediator of PCa cell motility and metastasis.

## RESULTS

### Trop-2 expression is increased in stages pT3/pT4 of human PCa

**Figure 1:**
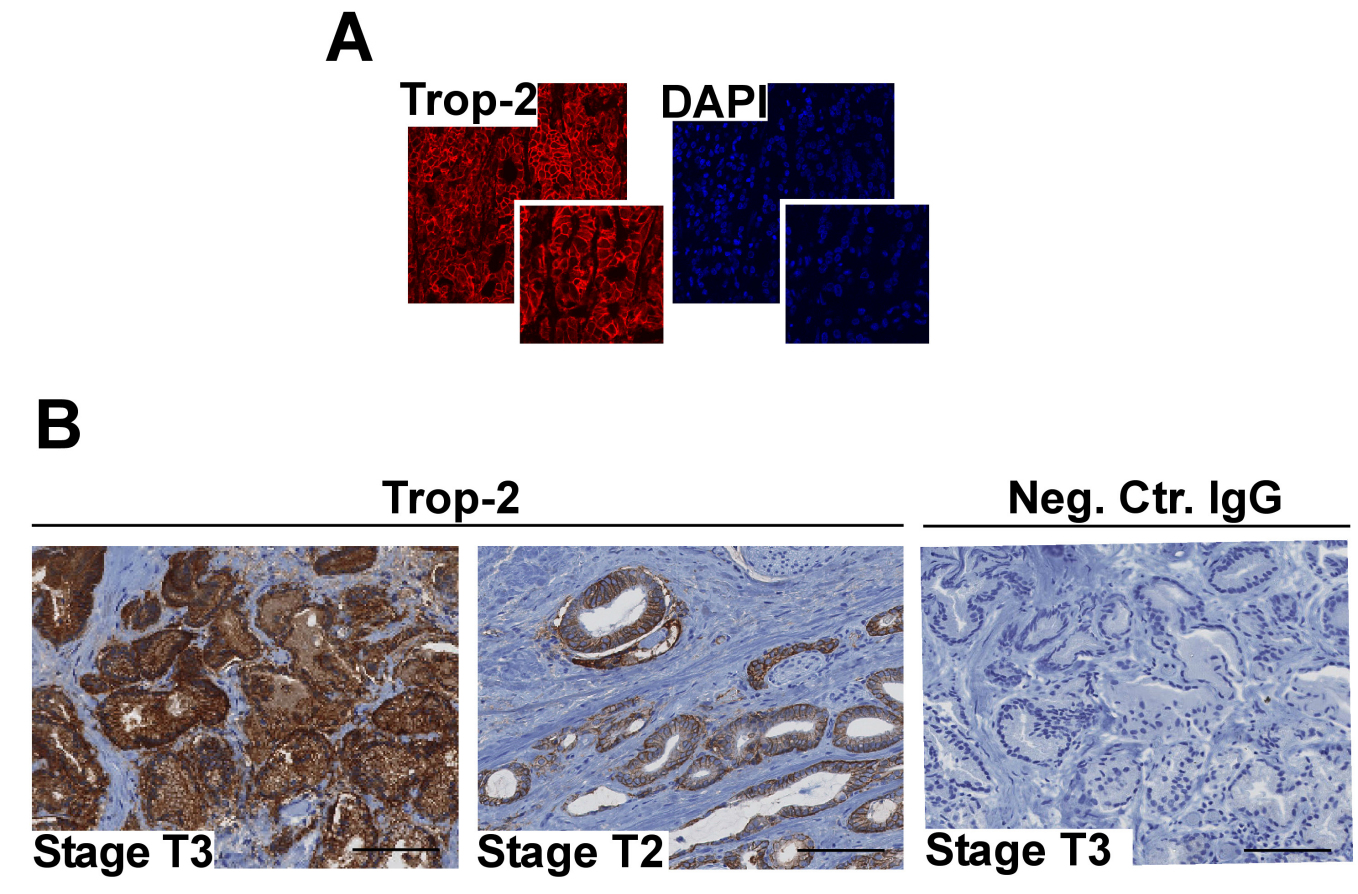
Trop-2 localization and expression in PCa **A.** Localization of Trop-2 as investigated by IF staining and confocal microscopy in human PCa (pT3 stage, Gleason Score 9). **B.** Representative IHC staining for Trop-2 using specimens from patients at pT3 (left) and pT2 (middle) stages of PCa is shown. A non-immune IgG was used as negative control on a stage pT3 section (right). Bars, 100 μm.

Our previous findings demonstrate a role of Trop-2 as an anti-adhesive and pro-migratory regulator in PCa [16, 17]. Here, we hypothesized that up-regulation of Trop-2 promotes escape of PCa cells from the primary tumor microenvironment and accelerates the onset of the metastatic cascade. Hence, we performed an immunofluorescence (IF) analysis of human PCa tissues; as depicted in Figure 1A, an abundant distribution of Trop-2 is found in membrane rims of the transformed cell population, whereas no reactivity is detected in the stromal compartment. We next analyzed Trop-2 expression levels in human PCa tissues by immunohistochemistry (IHC) using a Tissue Microarray (TMA) containing 104 cores from stage pT2 (organ-confined) and 44 cores from stages pT3/pT4 (PCa with extracapsular extension) cancer specimens collected from radical prostatectomies (Table 1). An example of Trop-2 expression in stages pT3 and pT2 of PCa is shown in Figure 1B. The expression of Trop-2 in these specimens was evaluated as low (IHC score <1.5) or high (IHC score ≥1.5) and is reported in Table 1. In these experiments, we observe strong expression of Trop-2 in 29.8% of pT2 stage and 81.8% in pT3/pT4 stage samples (Fisher's exact test *P* = 0.0002). We analyzed in parallel the frequency of cases with high Gleason score (8-10) in stage pT2 and stages pT3/pT4 and found that the differences are not statistically significant (Fisher's exact test *P* = 0.0940). Our results show that Trop-2 expression correlates with the stages pT3/pT4 in extracapsular invasive human PCa.

**Table 1:** Correlation of Trop-2 expression with pT3/pT4 in extracapsular invasive human prostate cancer

Score	pT2 (N = 104)	pT3/pT4 (N = 44)	*P*
	n (% of N)	n (% of N)	
Trop-2 IHC Score			0.0002
< 1.5	73 (70.2)	8 (18.2)	
≥ 1.5	31 (29.8)	36 (81.8)	
Gleason Score			0.0940
6-7	72 (69.2)	24 (54.6)	
8-10	32 (30.8)	20 (45.4)	

Expression of Trop-2 was evaluated by IHC using 148 PCa specimens as described in Methods. Samples were categorized in groups based on Trop-2 expression measured using IHC (< 1.5 and ≥ 1.5) or Gleason (6-7 and 8-10) scores. P, P value was determined as described in the Methods section.

### Trop-2 is up-regulated in prostate tumors of metastatic Transgenic Adenocarcinoma of Mouse Prostate (TRAMP) mice and forms a complex with the α5β1 integrin in PCa cells

**Figure 2:**
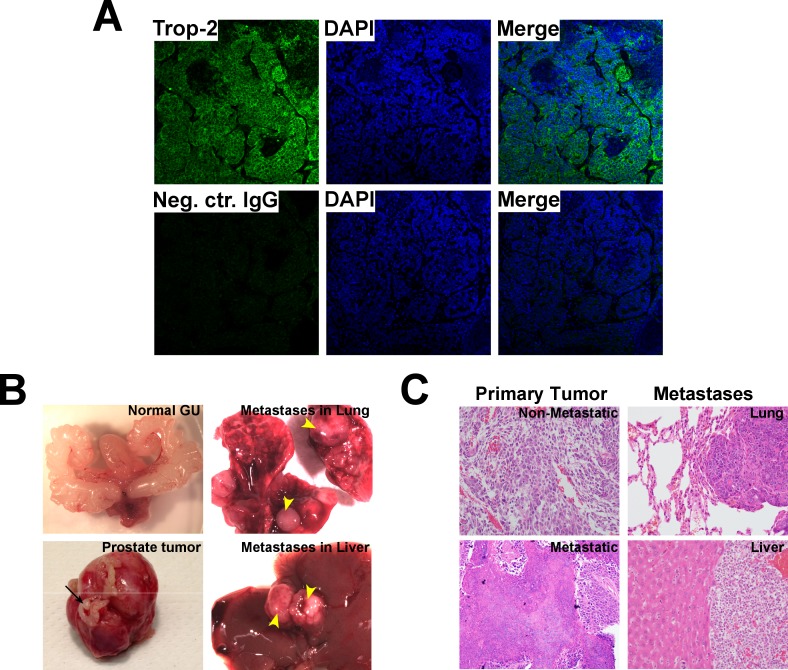
Analysis of Trop-2 expression in metastatic PCa from TRAMP mice **A.** IF analysis of Trop-2 expression in metastatic prostate tumors from TRAMP mice (top). Cell nuclei were counterstained with DAPI. A non-immune goat IgG was used as a negative control Ab (bottom). **B.** Representative images of a dissected normal genito-urinary (GU) system (top left), primary prostate tumor (bottom left), and lung (top right) and liver (bottom right) macroscopic metastases. Seminal vesicles (black arrow); metastases (yellow arrowheads). **C.** H&E staining of non-metastatic (top left), metastatic primary prostate tumors (bottom left), and of metastases in lungs (top right) and liver (bottom right).

Although mouse models of spontaneous PCa progression to metastasis are limited [22], the TRAMP model is known to develop aggressive and metastatic PCa [23]. We observe Trop-2 expression in metastatic prostate tumors of TRAMP mice using IF staining of prostate tumor tissue sections (Figure 2A). Macroscopic organ dissection of TRAMP mice (*n* = 69) was performed and primary tumors as well as metastases were analyzed. Figure 2B shows a representative normal genito-urinary (GU) (top left panel) and a primary tumor (bottom left panel), and also metastases in lung (top right panel) and liver (bottom right panel). Hematoxylin and Eosin (H&E) analysis of non-metastatic (top left panel) and metastatic (bottom left panel) primary tumors is shown in Figure 2C. Analysis of lung (Figure 2C, top right panel) and liver (Figure 2C, bottom right panel) metastases are also shown.

**Figure 3:**
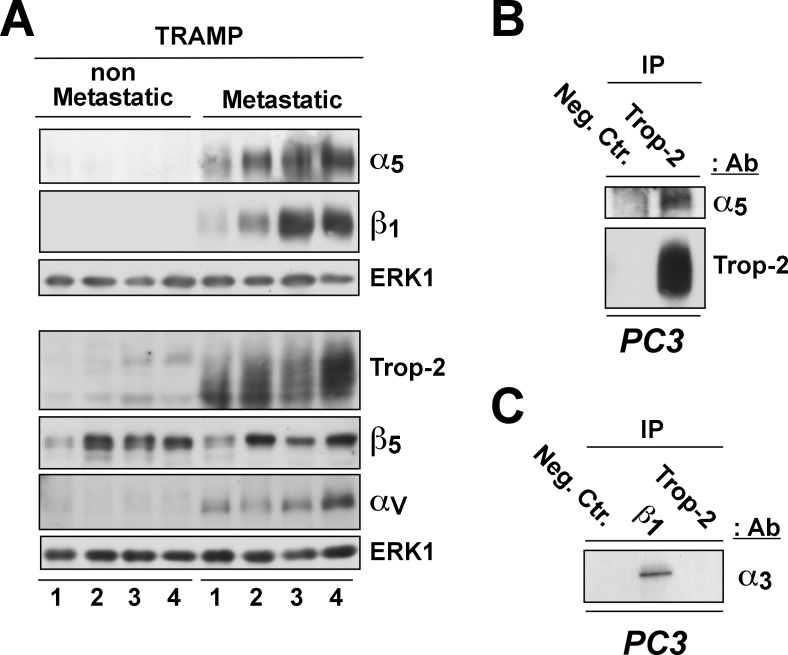
Correlation of Trop-2 and α5β1 integrin expression in murine PCa **A.** Analysis of α5, β1 (top), αv and β5 (bottom) integrin subunits, as well as of Trop-2 (bottom) expression by IB using protein lysates from non-metastatic (left) and metastatic (right) prostate tumors collected from TRAMP mice. ERK1, control of protein loading. **B.** Protein lysates of PC3 cells endogenously expressing Trop-2 were immunoprecipitated using an Ab targeting Trop-2; a non-immune mouse IgG was used as a negative control Ab (Neg. Ctr.). The immunoprecipitates were then separated by SDS-PAGE and analyzed by IB for detection of the α5 integrin subunit and Trop-2. **C.** Protein lysates of PC3 cells were immunoprecipitated using Abs targeting β1 integrins or Trop-2; a non-immune mouse IgG was used as a negative control Ab (Neg. Ctr.). The immunoprecipitates were then analyzed by IB for detection of the α3 integrin subunit.

We next analyzed the expression levels of Trop-2 and of α5, β1, β5, and αv integrin subunits in this experimental model, and compared metastatic (*n* = 4) with non-metastatic primary tumors (*n* = 4). As shown in Figure 3A, Trop-2 is highly expressed in metastatic tumor samples, but it is undetectable or expressed at low levels in non-metastatic tumors. Similarly, both α5 and β1 integrin subunits are strongly up-regulated in metastatic prostate tumors as compared with non-metastatic tumors. These changes are found to be specific, as β5, another integrin subunit, does not show appreciable variations in expression between metastatic and non-metastatic tumors. The αv integrin subunit, which does not associate with Trop-2 in PCa cells [16], was also preferentially expressed in metastatic tumors, suggesting the existence of additional regulatory mechanisms of this integrin subunit in PCa.

Since Trop-2 inhibits β1 integrin-mediated PCa cell adhesion to fibronectin (FN) [17] and induces migratory phenotypes on this ECM ligand [16], we tested the ability of Trop-2 to interact with α5β1. Co-immunoprecipitation experiments performed using PC3 human PCa cells demonstrate that Trop-2 specifically associates with the α5 integrin subunit (Figure 3B). In contrast, another β1-associated subunit, α3, does not interact with Trop-2 (Figure 3C). These results provide a biochemical basis for the ability of Trop-2 to specifically regulate α5β1 integrin functions.

### Trop-2 displaces focal adhesion kinase from focal contacts

**Figure 4:**
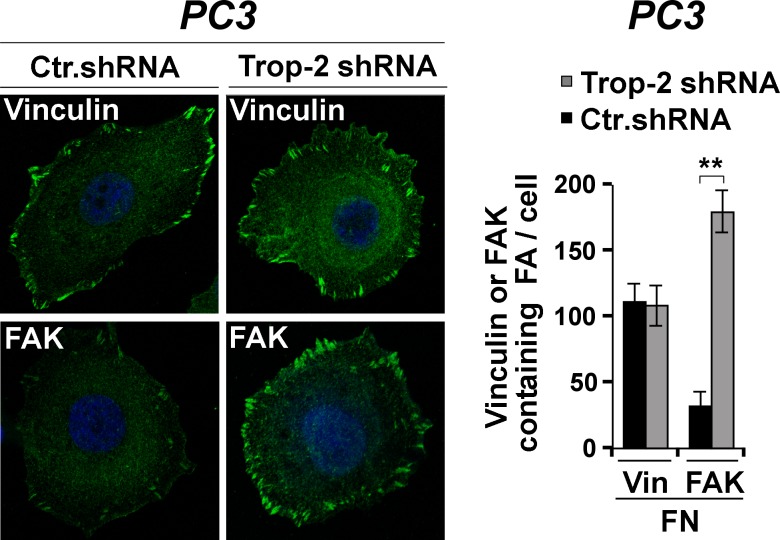
Trop-2-dependent modulation of FAK localization Localization of vinculin and FAK in PC3/Ctr.shRNA and PC3/Trop-2 shRNA cells seeded on FN was analyzed by IF (left). Vinculin (Vin)- and FAK-containing FAs were counted, and the average numbers per cell are shown in the bar graph (right). Error bars, SEM. **, Student's *t*-test *P* < 0.001.

Recent findings from our group have shown that Trop-2 inhibits accumulation of α5β1 integrin at FA sites [16], and promotes FAK activation [17]. To test this model, we stably silenced the expression of Trop-2 in PCa cells and looked at the dynamics of FAK subcellular distribution. As shown in Figure 4 (right panel), the average number of FAK-containing FA sites is found to be 178.60 ± 0.53 per cell in PC3/Trop-2 shRNA cells (FAK-containing FAs, *n* = 5,358/30 cells) as compared with 30.57 ± 0.4 per cell in PC3/control shRNA (Ctr.shRNA) cells (FAK-containing FAs, *n* = 917/30 cells). Conversely, the average number of vinculin-containing FAs is 107.87 ± 0.50 per cell in PC3/Trop-2 shRNA cells (FAs, *n* = 3,236/30 cells) as compared with 110.53 ± 0.43 per cell in PC3/Ctr.shRNA cells (FA, *n* = 3,316/30 cells), confirming the specificity of the observed response (Figure 4).

### Trop-2 is recruited in PCa exosomes that stimulate cell migration on FN

**Figure 5:**
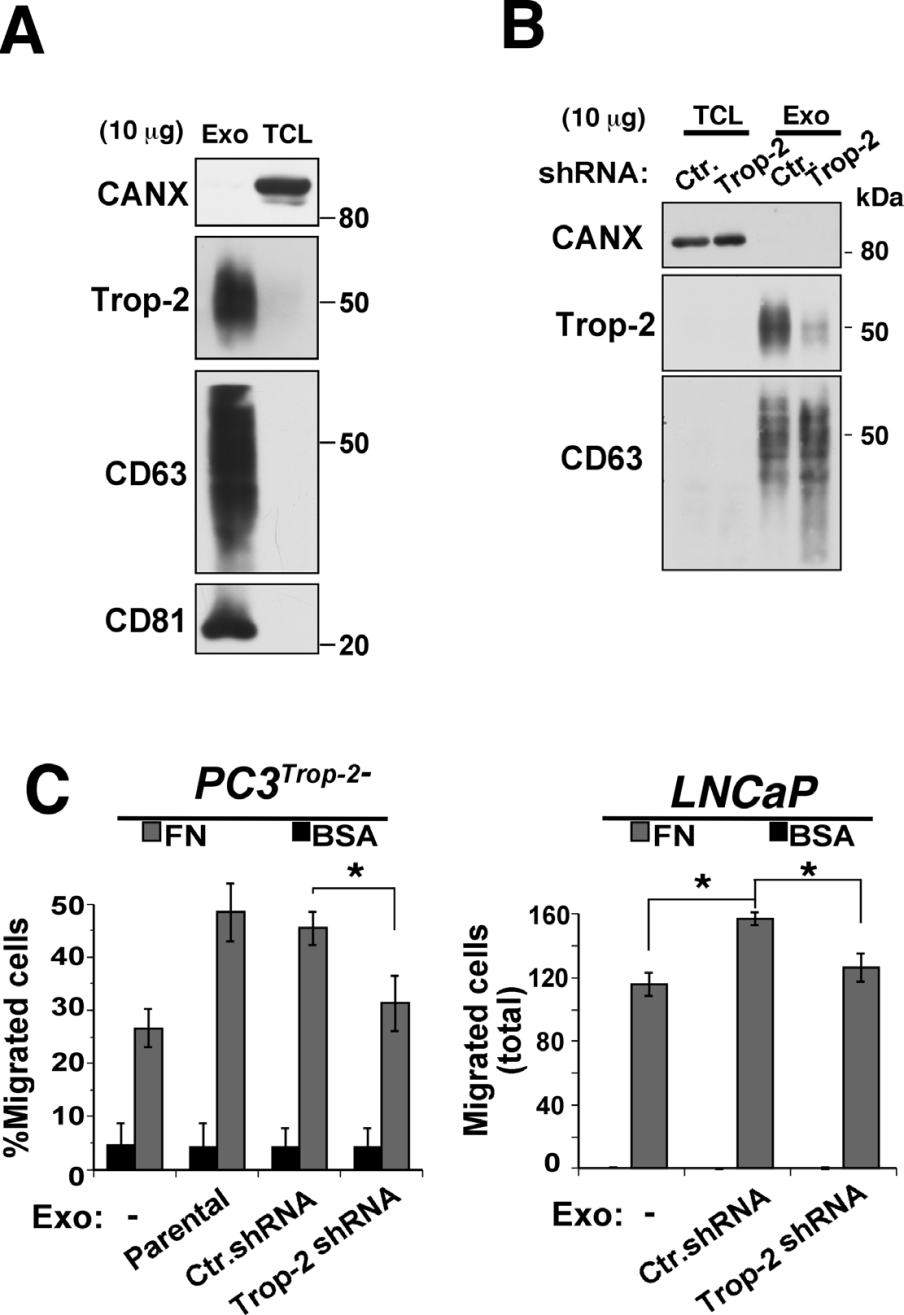
PC3 exosome uptake by PCa cells enhances cell migration on FN in a Trop-2-dependent manner **A.** Analysis of Trop-2 levels in purified PC3 exosomal lysates separated by SDS-PAGE in non-reducing conditions and immunoblotted using an Ab to Trop-2; CD63 and CD81 were used as positive exosomal markers while calnexin (CANX) was used as a negative marker for exosomes. Exo, exosomes; TCL, total cell lysates. **B.** IB analysis of Trop-2 expression in exosomes secreted by PC3 cells (Ctr.shRNA and Trop-2 shRNA) using an Ab to Trop-2; CD63 was used as positive exosomal markers while calnexin (CANX) was used as a negative marker for exosomes. Exo, exosomes; TCL, total cell lysates. **C.** Migration assays of PC3^Trop-2-^ (left) or LNCaP (right) cells either untreated or treated with 10 μg/ml of PC3 exosomes (Exo) in which Trop-2 is expressed (Parental and Ctr.shRNA) or down-regulated (Trop-2 shRNA). Left, χ^2^ test ; right, Student's *t*-test. *, P ≤ 0.05.

Release of exosomes from cancer cells has been shown to efficiently contribute to induction of metastatic dissemination by favoring intercellular communication [14, 24]. We hypothesized that Trop-2 may be recruited to these cellular compartments, where β1 integrins are also found [24-26]. Therefore, we isolated exosomes from PC3 culture supernatants and investigated by immunoblotting (IB) whether Trop-2 is recruited in these organelles as described in previous proteomic studies [27, 28]. Exosome preparations were characterized by IB analysis of exosomal markers: CD63, CD81 (Figure 5B); as control, IB analysis of Calnexin was performed to exclude contamination of endoplasmic membranes (Figure 5A). Our results show specific recruitment of Trop-2 in exosomes (Figure 5A). To investigate whether exosomal Trop-2 might affect cell migration on FN, we incubated Trop-2-negative cells, PC3^Trop-2-^ or LNCaP cells, with or without exosomes purified from PC3 cells which either express Trop-2 (Parental, Ctr.shRNA) or lack Trop-2 (Trop-2 shRNA). After confirming down-regulation of Trop-2 in exosomes, characterized by IB analysis of the exosomal marker CD63 (Figure 5B), we next performed migration assays FN as substrate. We show that 24 h treatment with Trop-2 containing exosomes increases cell migration on FN of both cell lines (Figure 5C). These findings suggest that a functional Trop-2/α5β1 integrin complex may accumulate in exosomes and stimulate migration of recipient cells.

## DISCUSSION

In this study, we demonstrate for the first time that Trop-2, an anti-adhesive and pro-migratory transmembrane protein, is up-regulated in human PCa with extracapsular extension (stages pT3/pT4) as compared to organ-confined (stage pT2) PCa, suggesting that this molecule plays a crucial role during cancer progression toward a metastatic phenotype. Mechanistically, we show that Trop-2 specifically binds the α5β1 integrin heterodimer and induces rearrangement of FA sites through displacement of FAK, thus perturbing the integrin signaling axis which is a major regulator of FA [29]. We finally find Trop-2 expression in exosomes secreted from PCa cells and demonstrate that Trop-2-containing exosomes stimulate migration of recipient Trop-2-negative cells on the α5β1 integrin substrate, FN.

The correlation between Trop-2 and disease progression suggests that this molecule is a novel biomarker of aggressive disease. During development, Trop-2 is expressed in the trophoblast, an actively invasive tissue at the interface between fetal and maternal circulation [30], whereas expression of this molecule in the adult is confined to a restricted number of tissues [21]. This pathway becomes exploited in malignancy, where Trop-2 is overexpressed in several human carcinomas, promotes accelerated tumor growth [21, 31], and correlates with unfavorable prognosis [18-20]. Our findings reinforce a role of this pathway in the progression from organ-confined to disseminated cancer and this phenotype appears to be more insightful than Gleason scores in the histopathological analysis of stages pT2 and pT3/pT4. It remains to be investigated whether Trop-2 overexpression may be predictive of biochemical recurrence after prostatectomy as Gleason scores are poor predictors of recurrence [32].

We find that the up-regulation of Trop-2 in metastatic tumors from TRAMP mice correlates with expression of β1 integrins (Figure 3A). While the mechanisms of Trop-2 up-regulation during disease progression are not known, we hypothesize that β1 integrins and Trop-2 expression may be co-regulated. Specifically, since previous studies from our group have shown that Insulin-like Growth Factor-1 Receptor (IGF-IR) plays a critical role in regulating β1 integrin expression and since the absolute levels of IGF-IR increase during PCa progression in TRAMP tumors [33-35], we propose that IGF-IR may be an upstream regulator of Trop-2/β1 integrin levels in metastasis-prone tumor types.

The proposed mechanism of metastatic dissemination, as suggested here, requires also up-regulation of the α5 integrin subunit expression and appears specific since it does not affect the α3 integrin subunit. The acquisition of invasive potential by transformed cells has been linked to loss of E-cadherin-dependent cell-cell contacts, epithelial-to-mesenchymal transition [36] and consequent up-regulation of α5 [7]. Consistently, we observe that α5 is overexpressed in metastatic versus non-metastatic PCa using TRAMP mice as a model of disease progression. This may involve, in addition to the previously reported ability of α5β1 to promote epithelial-to-mesenchymal transition during cancer progression [7], a direct role in leading PCa invasion through recognition sequences in FN for the α5 subunit [37]. Our previous findings that the reduced adhesive phenotype induced by Trop-2 results in a higher rate of cell migration on FN [16] are consistent with a model of Trop-2 modulation of α5β1 integrin signaling and selective displacement of FAK from FAs, that does not require changes in FAK expression as previously reported by our group [17].

We finally describe a novel mechanism involving accumulation of the Trop-2/β1 complex in secreted exosomes and demonstrate that exosomes containing Trop-2 impact neighboring tumor cells by enhancing their ability to migrate. These data suggest that therapeutics which interfere with the production, transfer or uptake of Trop-2-containing exosomes may attenuate tumor progression and metastasis.

Although more work is needed to fully elucidate the pro-invasive signaling pathways mediated by Trop-2 in PCa cells, the data presented here suggest that modulation of the Trop-2/α5β1 complex may provide new insights in the functional stratification of PCa patients with higher metastatic propensity and, therefore, in need of more aggressive treatments.

## MATERIALS AND METHODS

### Cells and culture conditions

Cell lines and transfectants, as well as culture conditions have been described previously [16, 17]. Authentication of the cell lines was provided with their purchase from American Type Culture Collection. PC3/Ctr.shRNA and PC3/Trop-2 shRNA cells were generated as described previously [16, 21].

### Mice

TRAMP mice, expressing SV40 large T antigen into the prostatic epithelium, were generated and characterized as described [23]. 23-54 week-old metastatic and non-metastatic TRAMP mice were used to isolate tumor samples and perform IB analysis. Distant sites were: liver and lungs. All mice were maintained under specific pathogen-free conditions. Care and handling of animals was in compliance with IACUC experimental protocols.

### Reagents and antibodies

The T16 mouse monoclonal antibody (mAb) against Trop-2 (gift of Dr. S. Alberti) and the TS2/16 mAb against β1 integrin (HB-243, ATCC) were used for IP. The following Abs were used for IB: mAbs against β1 integrin subunit (610468, BD Transduction Laboratories), CD63 (Ab8219, Abcam), CD81 (Ab23505, Abcam); goat polyclonal Abs (pAbs) against human and murine Trop-2 (AF650 and AF1122, R&D Biosystems); rabbit antisera against α3 and αv integrin subunits; rabbit pAbs against α5 integrin subunit (sc-10729, Santa Cruz Biotechnology), β5 integrin subunit (AB1926, Millipore), Calnexin (sc-11397, Santa Cruz Biotechnology) and ERK1/2 (sc-93, Santa Cruz Biotechnology). The goat pAb against human Trop-2 (AF650, R&D Biosystems), a mAb to Vinculin (MAB1624, Millipore) and a mAb to FAK (05-537, Millipore) were used for IF. A goat pAb against human Trop-2 (R&D Biosystems) was used for IHC. Non-immune goat IgG (SantaCruz), non-immune rabbit IgG (Sigma) and non-immune mouse IgG (Pierce) were used as negative control Abs. FN purification from human plasma has been previously described [17].

### Immunohistochemical analysis

TMAs were constructed at the Vancouver Prostate Cancer Centre (Vancouver, Canada) from 74 men with newly diagnosed, previously untreated, clinically localized high-risk PCa, who underwent radical prostatectomy at the same Center. Ethical approval was obtained from the Institutional Ethical Review Board.

Specimens were identified for benign and cancerous sites and marked in donor paraffin blocks using matching H&E-stained reference slides. The TMAs were constructed using a manual tissue microarrayer (Beecher Instruments, Silver Spring, MD, USA). Each marked block for benign and cancerous sites was sampled two times with a core diameter of 0.6 mm arrayed in a rectangular pattern with 1 mm between the center of each core, creating a duplicate TMA layout with a total of 148 cores. The TMA paraffin block was sectioned into 5-μm sections and mounted on positively charged slides.

IHC staining was conducted using a goat pAb to Trop-2 (AF650, R&D Biosystems, dilution 1:25) by a Ventana autostainer model Discover XTTM (Ventana Medical System, Tuscan, AZ, USA) with an enzyme-labelled biotin-streptavidin system and solvent-resistant 3,3′-diaminobenzidine Map kit. The slides were scanned on a BLISS system (Bacus Laboratory, North Lombard, IL, USA) and scored from 0 to +3 by a pathologist (L.F.) based on the staining intensity and the proportion of cells stained. Normal goat IgG was used as negative control Ab. All comparisons of staining intensities were made at 200X magnifications.

### Immunofluorescence and confocal microscopy

Antigen retrieval was performed on rehydrated formalin-fixed paraffin-embedded sections from human or TRAMP mice PCa samples by incubation in 10 mM Sodium Citrate Buffer (pH 6.0) at 95°C for 23 min. The sections were blocked for 1 h at room temperature with PBS / 5% BSA. Staining was performed by incubation of tissue samples with primary Abs (1:100) for 1 h at room temperature, followed by incubation with Alexa Fluor 633-Donkey anti goat (1:250) for 20 min at room temperature. Nuclei were counterstained using DAPI. After three washes, coverslips were mounted on the sections using Pro-Long anti-fade reagent (Invitrogen), and slides were analyzed on an inverted confocal microscope (LSM510, Carl Zeiss). Immunofluorescence analysis of PC3 cells using Alexa Fluor 488 goat anti-mouse (1:250) for 60 min at room temperature was performed as described [16].

### Generation of tumor lysates

Tumor lysates were prepared by homogenizing the tissues on ice using the following lysis buffer: 100 mM Tris-HCl (pH 7.4), 150 mM NaCl, 5% SDS, 0.1% Triton X-100, 1 mM benzamidine, 10 μg/mL Soybean Trypsin Inhibitor, 10 μg/mL leupeptin, 1 mM PMSF, 1 μg/mL pepstatin A, and 1 μM calpain inhibitor. The lysates were boiled for 5 min and centrifuged at 13,000 rpm for 20 min. Supernatants were collected and protein content was determined using the DC Protein Assay Kit (Bio-Rad). The protein samples (50 μg per lane) were separated by SDS-PAGE and transferred onto PVDF membranes for IB.

### Isolation and immunoblotting analysis of PCa exosomes

Exosomes were isolated from PCa cells as described [24, 38]. Purified exosomes were lysed with radioimmunoprecipitation assay buffer (10 mM Tris-HCl at pH 7.4, 150 mM NaCl, 1 mM EDTA, 0.1% SDS, 1% Triton X-100, and 1% sodium deoxycholate) supplemented with protease inhibitors. The protein samples (10 μg per lane) were separated by SDS-PAGE under non-reducing conditions and transferred onto PVDF membranes for IB.

### Immunoprecipitation

To collect nuclear and cytoplasmic fractions, cells were washed with cold PBS and lysed by scraping in 20 mM Tris–HCl (pH 7.4), 150 mM NaCl, 1mM CaCl_2_, 1mM MgCl_2_, 1% NP-40, 1 mM benzamidine, 10 μg/ml leupeptin, 1 mM PMSF, 1 μg/ml pepstatin A, 1 μM calpain inhibitor, 1mM Na_3_VO_4_, 1 mM Na_4_O_7_P_2_. The cells were subjected to 3 cycles of sonication (15 sec each) on ice. After 15 min incubation on ice, lysates were centrifuged at 12,000g for 10 min, and supernatants were collected and pre-cleared by two consecutive incubations with protein G-Sepharose at 4ºC for 45 min. Binding to specific Abs was performed by incubation at 4ºC for 3 h, followed by incubation with protein G-Sepharose at 4ºC for 1 h. After six washes with lysis buffer, immunocomplexes were eluted with 100 mM glycine pH 2.5, followed by pH neutralization using Tris to a final concentration of 50 mM. The immunocomplexes were then separated by SDS-PAGE, transferred onto PVDF membrane, and subjected to analysis by IB.

### Exosome treatment and migration assay

Cell treatment with exosomes was performed as previously described [24]. Briefly, LNCaP and PC3^Trop-2-^ cells were serum starved for 18 h, then treated for 24 h with 20 μg/ml of exosomes obtained from PC3 cells. The cells treated with PC3-derived exosomes were trypsinized, extensively washed with PBS and subsequently plated to perform the migration assay. Cell migration assays on FN has been performed as previously described using Millicell inserts (Millipore) with 8 (for LNCaP) or 12 μm (for PC3^Trop-2-^) pores [16]. Briefly, chambers were coated on both top and bottom layers with FN (10 μg/mL) or 1% BSA overnight at 4ºC. After cell detachment and trypsin inactivation, cells were seeded on coated transwell chambers at 37°C for 6 h. After fixation with 3.7% paraformaldehyde (PFA), cells attached on both layers of the porous filter were stained with 1 μg/mL 4′,6-diamidino-2-phenylindole (DAPI) and pictures of nuclei were acquired by fluorescence microscopy. Then, cells on the top layer were removed using a cotton swab, and pictures of nuclei from cells migrated to the bottom layer were acquired. For each group of PC3^Trop-2-^ cells treated with or without exosomes, the ratio between number of cells migrated onto the bottom layer and total (top + bottom) number of cells attached on the filter was calculated. For LNCaP cells, only the total number of cells attached on the bottom layer was calculated.

### Statistical analysis

For patients' samples, Fisher's exact test was used to examine the association between dichotomized biomarkers (Trop-2) and PCa stage (Gleason score). For FA analysis, *t*-test was used to evaluate the average numbers of FA per cell between PC3/Ctr.shRNA and PC3/Trop-2 shRNA cells. For migration assays χ^2^ tests and *t*-test were used to compare the migration between treatments. Stata 12.0 (StatCORP LP, College Station, TX, USA was used for data analysis *P* ≤ 0.05 was considered as statistical significance.

## Abbreviations

PCa: prostate cancer
ECM: extracellular matrix
TNM: tumor, node and metastasis
PSA: prostate specific antigen
TRAMP: TRansgenic Adenocarcinoma of Mouse Prostate
IB: immunoblotting
mAb: monoclonal antibody
pAb: polyclonal antibody
IF: immunofluorescence
IHC: immunohistochemistry
TMA: Tissue Microarray
FAK: focal adhesion kinase
FN: fibronectin
