# Erratum: Trop-2 is up-regulated in invasive prostate cancer and displaces FAK from focal contacts

**DOI:** 10.18632/oncotarget.6187

**Published:** 2015-10-20

**Authors:** Marco Trerotola, Kirat K. Ganguly, Ladan Fazli, Carmine Fedele, Huimin Lu, Anindita Dutta, Qin Liu, Tiziana De Angelis, Luke W. Riddell, Natalia A. Riobo, Martin E. Gleave, Amina Zoubeidi, Richard G. Pestell, Dario C. Altieri, Lucia R. Languino

***Oncotarget. 2015; 16: 14318-14328***

**PMID:** 26015409

**DOI:** 10.18632/oncotarget.3960

Present:

**GRANT SUPPORT**

This work was supported by NIH (LRL, DCA), NIH-R01CA109874 (LRL), NIH-R01CA089720 (LRL), NIHR01CA086072 (RGP); the Office of the Assistant Secretary of Defense for Health Affairs through the Prostate Cancer Research Program under Award No. W81XWH-13-1-0193 (DCA), and by a Post-Doctoral Research Fellowship from the Italian Association for Cancer Research to M.T. and by a Post-Doctoral Research Fellowship from the American Italian Cancer Foundation to C.F. Research in this publication includes work carried out using the Sidney Kimmel Cancer Center Bioimaging Facility and the Translational Research & Pathology Shared Resource, which are supported in part by NCI Cancer Center Support Grant P30 CA56036 (RGP). This project is also funded, in part, under a Commonwealth University Research Enhancement Program grant with the Pennsylvania Department of Health (H.R.); the Department specifically disclaims responsibility for any analyses, interpretations or conclusions.

Corrected:

**GRANT SUPPORT**

This work was supported by NIH (LRL, DCA), **NIH-P01140043**, NIH-R01CA109874 (LRL), NIH-R01CA089720 (LRL), NIH-R01CA086072 (RGP); the Office of the Assistant Secretary of Defense for Health Affairs through the Prostate Cancer Research Program under Award No. W81XWH-13-1-0193 (DCA), and by a Post-Doctoral Research Fellowship from the Italian Association for Cancer Research to M.T. and by a Post-Doctoral Research Fellowship from the American Italian Cancer Foundation to C.F. Research in this publication includes work carried out using the Sidney Kimmel Cancer Center Bioimaging Facility and the Translational Research & Pathology Shared Resource, which are supported in part by NCI Cancer Center Support Grant P30 CA56036 (RGP). This project is also funded, in part, under a Commonwealth University Research Enhancement Program grant with the Pennsylvania Department of Health (H.R.); the Department specifically disclaims responsibility for any analyses, interpretations or conclusions.

